# Measurement of Ability in Adaptive Learning and Assessment Systems when Learners Use On-Demand Hints

**DOI:** 10.1177/01466216221084208

**Published:** 2022-04-18

**Authors:** Maria Bolsinova, Benjamin Deonovic, Meirav Arieli-Attali, Burr Settles, Masato Hagiwara, Gunter Maris

**Affiliations:** 1 7899 Tilburg University, Tilburg, Netherlands; 2 501878 Corteva Agriscience, Johnston, IA, USA; 3 145134 Center for Educational Technology, Tel Aviv, Israel; 4 Duolingo, Pittsburg, PA, USA; 5 Octanove Labs LLC, Seattle, WA, USA; 6 Tata Consultancy Services, Zaventhem, Belgium

**Keywords:** adaptive learning systems, item response tree models, measurement, multidimensional nominal response model, hints, item response theory, measurement theory

## Abstract

Adaptive learning and assessment systems support learners in acquiring knowledge and skills in a particular domain. The learners’ progress is monitored through them solving items matching their level and aiming at specific learning goals. Scaffolding and providing learners with hints are powerful tools in helping the learning process. One way of introducing hints is to make hint use the choice of the student. When the learner is certain of their response, they answer without hints, but if the learner is not certain or does not know how to approach the item they can request a hint. We develop measurement models for applications where such on-demand hints are available. Such models take into account that hint use may be informative of ability, but at the same time may be influenced by other individual characteristics. Two modeling strategies are considered: (1) The measurement model is based on a scoring rule for ability which includes both response accuracy and hint use. (2) The choice to use hints and response accuracy conditional on this choice are modeled jointly using Item Response Tree models. The properties of different models and their implications are discussed. An application to data from Duolingo, an adaptive language learning system, is presented. Here, the best model is the scoring-rule-based model with full credit for correct responses without hints, partial credit for correct responses with hints, and no credit for all incorrect responses. The second dimension in the model accounts for the individual differences in the tendency to use hints.

## Introduction

Adaptive learning and assessment systems are designed to dynamically adjust the level or type of practice and instruction material based on an individual learner’s abilities or skills and other characteristics. In such systems, accurate and precise measurement of the learners’ level of ability is important since monitoring the development of learners’ skills is crucial to adapt the learning material to their level. The level of learners’ ability is usually estimated based on the correctness of the responses that they give to the practice items. However, information on learners’ behavior in the system, and in particular whether learners use hints, can be used to obtain additional information about the measured abilities or skills, and can be included in the scoring rule when estimating ability. Hint use can be considered as process data as opposed to the response accuracy which constitutes product data from the assessment. While a large number of models have been developed to incorporate different types of process data into measurement, for example response times (for an overview see e.g., Kyllonen & Zu, 2016), measurement models for applications in which respondents may request hints instead of directly answering items could be more fully developed and explored.

Previous research on modeling hint use of learners yield different approaches. One of the early works by Aleven and colleagues (e.g., Aleven & Koedinger, 2000; Aleven et al., 2004) took the approach of predefining productive rules for help-seeking behavior (i.e., the ideal behavior) and examined whether learners adhere to these rules. This normative model of help-seeking suggests that there are appropriate times for requesting hints. In some conditions using hints including bottom-out-hints that show the correct response is warranted, particularly when students are at a low level of skill acquisition. The authors found that the majority of learners (72%) did not follow these productive rules, but rather exhibit what they called “unproductive behavior” and “hint abuse,” where learners used the hints to get at the answer rather than to learn. It may be noted, however, that using bottom-out hints does not necessarily have to be considered unproductive, as they can also provide new learners with worked examples of how to solve problems (see Shih et al., 2011, for a model that distinguishes productive use of bottom-out hints from hint abuse by means of response times).

A different approach was taken by Feng and Heffernan (2010), Wang and Heffernan (2011), and Wang et al. (2010). In their research, a data-driven approach was taken without predetermined assumptions about hint use, except to assume that the more hints used by the learner, the lower the probability that they possess the knowledge. The model they proposed—the Assistance Model (AM; Wang & Heffernan, 2011)—uses the information about the number of hints used and the number of answer attempts till correct to predict the performance of learners on the next item. Since the AM seemed to predict the performance on the next item, the researchers added the model to other models that track learning, combined with a Bayesian Knowledge Tracing model (Wang & Heffernan, 2011) and a partial credit model (Wang et al., 2010).

When modeling hint use behavior, Goldin et al. (2013) applied a multinomial logistic regression that distinguishes hint-request tendency from proficiency. They found that seeking just one hint is associated with repeated hint-seeking, but when students do make attempts to solve a problem after viewing a hint, they succeed about half of the time. They also found that the individual differences in the tendency to use hints are stronger than the differences in proficiency.

The approaches to modeling hint use discussed so far focus on the prediction of the correctness of the next response. Another perspective that has been taken by different authors (e.g., Cen et al., 2006; Ueno & Miyazawa, 2017) and is considered in this paper focuses on the measurement of ability in the presence of hints. For measurement of ability, item response theory (IRT) models are often used (for an overview see e.g., Hambleton & Swaminathan, 2013; van der Linden & Hambleton, 2013). These are statistical models that relate the probability of a correct response to an item to the persons’ ability, which allows the estimation of the level of ability given the observed responses. One of the most common IRT models is the two-parameter logistic model (2PL; Birnbaum, 1968) in which the probability of a correct response to an item is modeled as a function of the person’s ability and the item characteristics:

(1) $$\text{Pr}\left(X_{i}=1|\theta \right)=\frac{\exp\left(\alpha_{i}\left(\theta -\delta_{i}\right)\right)}{1+\exp\left(\alpha_{i}\left(\theta -\delta_{i}\right)\right)},$$

where *X*_*i*_ is the binary response accuracy on item *i* (1—correct, 0—incorrect), *θ* is the ability latent variable, *δ*_*i*_ is the difficulty of the item, and *α*_*i*_ is the discrimination of the item. The response accuracies on different items are assumed to be conditionally independent given the latent variable. The density of the data from *N* learners to *K* items is the following:

(2) $$f\left(\alpha \text{,}\delta \text{;}x\text{,}z\right)\;=\prod_{p}\int \prod_{i}{\left(\frac{\exp\left(x_{pi}\alpha_{i}\left(\theta -\delta_{i}\right)\right)}{1+\exp\left(\alpha_{i}\left(\theta -\delta_{i}\right)\right)}\right)}^{z_{pi}}N\left(\theta ;0,1\right)d\theta ,$$

where **x** is the partially complete data matrix of response accuracy with *x*_*pi*_ being the response accuracy of person *p* to item *i*, **z** is the *N* × *K* matrix with each element *z*_*pi*_ indicating whether learner *p* responded to item *i* (1—yes, 0—no), ***α*** and ***δ*** are the vectors of length *K* of the item discrimination and difficulty parameters, and the ability is assumed to have a normal distribution in the population of learners with the mean and variance constrained to 0 and 1 for identification purposes.

When in an adaptive learning environment hints are provided to learners, the standard measurement model needs to be adapted to include the hint use. When adapting the measurement model, it is important to distinguish the two ways in which hints can be presented to learners: (1) providing hints after an incorrect response, or (2) providing hints on-demand. These two ways of providing hints differ both from the learning perspective and with respect to how hints should be incorporated in the measurement model. The differential learning effect of providing hints after an incorrect response versus on-demand has been studied by Razzaq and Heffernan (2010). They found that the students learned reliably more with hints on-demand than with hints after an incorrect response. On-demand hints might also be preferable as a tool for improving the learners’ motivation by providing them with more control of their learning process and giving them more freedom within the learning environment which might be advantageous for the learning process.

The different ways of providing hints also require different statistical treatment. Let us by *y*_*pi*_ denote a binary indicator of whether the learner *p* was provided with a hint when responding to item *i*. When a hint is provided after an incorrect response, the hint use itself is not a random variable since *y*_*pi*_ = 1 − *x*_*pi*1_ where *x*_*pi*1_ is the response accuracy on the first attempt to answer item *i*. That is, if the response is correct on the first attempt the hint is never provided, while if the response on the first attempt is incorrect then the hint is always provided. To include this in a measurement model, one can instead of modeling the distribution of the binary response accuracy as in the 2PL model consider the distribution of the accuracy variable with three levels: $X_{i}^{*}=2$ if the response is correct after the first attempt, $X_{i}^{*}=1$ if the response is incorrect after the first attempt, but correct after the hint was provided in the second attempt, and $X_{i}^{*}=0$ if the response is incorrect in both attempts. The distribution of $X_{i}^{*}$ given the ability latent variable can be modeled using IRT models for polytomous data, for example the partial credit model (Masters, 1982) or the graded response model (Samejima, 1969), as has been done by Ueno and Miyazawa (2017).

When hints are provided on demand, the hint use *Y*_*i*_ on item *i* is itself a random variable, since the learner is always free to choose whether to request a hint or not. In this case, instead of having one variable $X_{i}^{*}$ with three possible outcomes, for each item there are two binary random variables (*X*_*i*_ and *Y*_*i*_) with four possible outcomes in total. One approach for dealing with this is to treat the use of hints similar to incorrect responses. Then, a variable $X_{i}^{**}=ℐ\left(X_{i}=1\right)ℐ\left(Y_{i}=0\right)$ (i.e., 1 if the response is correct without a hint, and 0 otherwise) can be modeled using dichotomous IRT models or their generalizations like the Additive Factors Model (Cen et al., 2006). An alternative, which we are going to pursue in this paper, is to model the full joint distribution of *X*_*i*_ and *Y*_*i*_ without collapsing the different types of outcomes. Our motivation for this is that not only the accuracy of the responses but also whether students use hints may be informative about ability. At the same time, there might be other individual differences between learners with respect to hint use behavior that are not directly related to ability, which should also be taken into account when modeling the joint distribution of response accuracy and hint use. The goal of this paper is to develop measurement models for ability for applications where hints are provided on-demand that allow one to estimate learner’s level of ability based on both response accuracy and hint use behavior but at the same time acknowledge that there might be other relevant differences between the learners influencing their interactions with the learning system.

The rest of the paper is organized as follows. The *Data* section describes the adaptive language learning system Duolingo, data from which is used to illustrate the different approaches to the joint modeling of response accuracy and hint use. The details on the dataset used in the study are provided. In the section *Jointly modeling hint use and response accuracy*, we propose different modeling approaches for measurement of ability when hints are available on-demand. The *Methods* section describes the data analysis methods. The *Results and Discussion* section presents and discusses the results of applying different measurement models to the data from Duolingo. The paper ends with general conclusions.

## Data

Data with hint use was obtained from Duolingo, an online language learning platform with more than 200 million registered users. A Duolingo course is organized into a tree-like structure called a skill tree. A node in this tree is called a skill. A row in a course is a set of skills which have the same depth in the skill tree. Users can move on to the next row only after they complete all skills in the previous row. After starting a lesson, users learn the language by solving a series of exercises (i.e., items) called challenges. A consecutive series of challenges is called a session. Users can also choose to practice the skill they already completed, by pressing a practice button on a skill page. This is called a skill practice session, which shows one a series of challenges on the words and grammar that need reviewing the most according to Duolingo’s internal word strength model.

The dataset from Duolingo available for analysis contained items completed by learners between November 9, 2015, and December 8, 2015. Only users who created a new account during the data collection period and reached the tenth row in their respective course’s skill tree were included in the sample. From the six language courses that were available for analysis, two (with the largest number of learners) were included in this study. One dataset (from the course for learning Spanish for English speakers, i.e., Spanish-from-English) was used for preliminary analysis to get insights about what the important characteristics of the data are which should be taken into account when developing the models for hint use and response accuracy. Another dataset (English-from-Portuguese) was used after the models were developed to select the model best describing the data.

For data analysis subsets of the data were selected. First, for each of the two courses only data from a single platform (with the largest number of users) was selected to avoid the additional differences between the learners from the different user interfaces on different platforms: data from Android was used in the Spanish-from-English course, and data from iOS was used for the English-from-Portuguese course. Second, only challenges which were the translate challenge (from foreign language to native language) were kept and only the first time a user responded to this challenge was used. These translation items give the learners the opportunity to ask for a hint by checking the translation of one or more words in the sentence. The learner may hover over any word in the sentence and see the translation to their native language. If a learner hovers over at least one of the words, this is registered as a learner using a hint on an item (*Y*_*pi*_ = 1). The learner has to type their response and the accuracy of the response is recorded. The item is considered correct if the sentence is translated correctly.^1^

The subset of users and items included 2639 learners and 8848 items for Spanish-from-English, and 5264 learners and 7087 items for English-from-Portuguese. Some additional selection criteria were applied to the learners and the items. Only the items which are complete sentences (i.e., not word combinations or separate words) and have more than two non-article words were included. Items with less than 40% observed responses were removed. For the remaining items, users who had more than 10 responses were selected. Finally, to make sure that there is not a lot of overlap in words between the selected sentences, for each item pair for which there was more than 70% overlap in words the shortest item was removed. This resulted in 89 items and 1109 users for Spanish-from-English and 99 items and 3845 learners for English-from-Portuguese.

## Jointly modeling hint use and response accuracy

### Initial model: Hint use as part of the scoring rule

Information on whether learners use hints can be considered process data as opposed to the response accuracy which constitutes product data from the assessment. Process data can be used to obtain additional information about the measured abilities or skills, and can be included in the scoring rule when estimating ability. This has been done in the context of response times, which is another example of process data. Maris and van der Maas (2012) proposed a model for jointly modeling response accuracy and response time called signed-residual-time model in which the contribution of response time in the item score depends on the accuracy of the response:

(3) $$S=\sum_{i}\left(2X_{i}-1\right)\left(d-T_{i}\right),$$

where *S* is the total score of a person, *X*_*i*_ is response accuracy on item *i*, *T*_*i*_ is response time, and *d* is the time limit. The remaining time before the deadline is either added to the score if the response is correct, or subtracted from the score if the response is incorrect. In this way, the scoring rule encourages fast correct responses, but discourages fast incorrect responses. An IRT model has been developed based on this scoring rule and the assumption of conditional independence between the scores on different items given ability, which allows to estimate the differences between the items in their difficulty and the differences between the persons in their ability based both on their response accuracy and response time.

The same idea can be used in the context of hint use. The contribution of the item to the score can be based on both whether the response was correct and on whether it was obtained with a hint. With two binary variables (*X*_*i*_ and *Y*_*i*_), four different outcomes can be considered, each matching an item score:

(4) $$S_{pi}=\{\begin{array}{l} 0\text{ if }X_{pi}=0,Y_{pi}=0;\\ 1\text{ if }X_{pi}=0,Y_{pi}=1;\\ 2\text{ if }X_{pi}=1,Y_{pi}=1;\\ 3\text{ if }X_{pi}=1,Y_{pi}=0. \end{array}$$

Higher scores (2 and 3) are given to correct responses, and the impact of hint use on the item score depends on whether the response is correct (similarly to the differential contribution of response time in the signed-residual-time model): If the response is correct, then a higher score is obtained when no hints were used, while if the response is incorrect, then a higher score is obtained when a hint was used. Similarly to how response time is treated in the signed-residual-time model, here correct responses without hints are encouraged, while incorrect responses without hints are discouraged.

An IRT model can be derived from this scoring rule for which the total score for the person *∑*_*i*_*S*_*pi*_ is a sufficient statistic for the person’s ability, and *∑*_*p*_*S*_*pi*_ is the sufficient statistic for the item’s difficulty (see Appendix for the details of the derivation):

(5) $$\text{Pr}\left(S_{i}=s|\theta \right)=\frac{\exp\left(s\left(\theta -\delta_{i}\right)\right)}{\sum_{r=0}^{3}\exp\left(r\left(\theta -\delta_{i}\right)\right)},$$

where *s* ∈ {0, 1, 2, 3}, *θ* is ability latent variable, and *δ*_*i*_ is the difficulty of item *i*. This is a constrained version of the partial credit model in which there is a single item difficulty parameter instead of multiple threshold parameters. This single parameter can be interpreted as follows: When ability of a person is equal to its value then all four outcomes are equally likely. The higher *δ*_*i*_ is, the higher the expected score on the item is.

### Extending the model

The model in Equation 5 implies that the items do not differ in their discriminatory power, that is the strength of the relationship between the item score and ability is assumed to be the same across items. However, it might be beneficial to extend the model for hint use and accuracy to allow for the items to differ in the strength of the relationship between the item score and ability, similarly to how the Rasch model (Rasch, 1960) has been extended to the 2PL model and the signed-residual-time model has been extended to its generalized version (van Rijn & Ali, 2018). The extended model is:

(6) $$\text{Pr}\left(S_{i}=s|\theta \right)=\frac{\exp\left(s\alpha_{i}\left(\theta -\delta_{i}\right)\right)}{\sum_{r=0}^{3}\exp\left(r\alpha_{i}\left(\theta -\delta_{i}\right)\right)},$$

where *α*_*i*_ > 0 is the discrimination parameter of item *i*.

**Figure 1.**
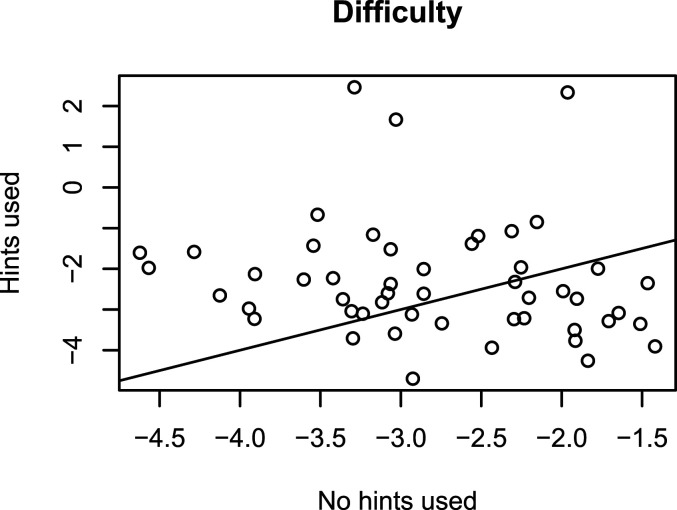
Item difficulties in the Duolingo Spanish-from-English data set estimated separately from the responses in which no hints were used (on the *x*-axis) and from the responses in which hints were used (on the *y*-axis). The parameters were estimated for the two-parameter logistic model using marginal maximum likelihood and the R-package mirt (Chalmers, 2012). The estimates do not lie on the identity line, which means that it is not reasonable to assume that the difficulty of the items is independent on hint use.

The model in Equation 6 implies the following conditional distributions of response accuracy given hint use:

(7) $$\text{Pr}\left(X_{i}=1|\theta ,Y_{i}=1\right)=\text{Pr}\left(S_{i}=2|\theta ,S_{i}\in \left\{1,2\right\}\right)=\frac{\exp\left(\alpha_{i}\left(\theta -\delta_{i}\right)\right)}{1+\exp\left(\alpha_{i}\left(\theta -\delta_{i}\right)\right)}$$

for when the hints were used, and

(8) $$\text{Pr}\left(X_{i}=1|\theta ,Y_{i}=0\right)=\text{Pr}\left(S_{i}=3|\theta ,S_{i}\in \left\{0,3\right\}\right)=\frac{\exp\left(3\alpha_{i}\left(\theta -\delta_{i}\right)\right)}{1+\exp\left(3\alpha_{i}\left(\theta -\delta_{i}\right)\right)}$$

for when the hints were not used. In both cases, it is a 2PL model with the same difficulty (*δ*_*i*_), but different discrimination (*α*_*i*_ and 3*α*_*i*_, respectively). Note, that the difficulty of item *i* can be interpreted as such a point on ability scale for which the probabilities of obtaining the four different scores on the item are all equal to 1/4 . Thus, the model poses a constraint on the conditional difficulties of the items. To evaluate whether such a constraint makes sense in the context of Duolingo data, we fitted the 2PL model separately to the Spanish-from-English accuracy data of those responses where hints were used, and those responses where hints were not used. Figure 1 shows the estimated conditional difficulties of the items. Since the dots do not lie on the identity line, we conclude that it might be beneficial to extend the model to allow for the conditional accuracy models to have separate difficulty parameters. The model can be extended as follows:

(9) $$\text{Pr}\left(S_{pi}=s|\theta \right)=\frac{\exp\left(s\alpha_{i}\theta +\delta_{is}\right)}{\sum_{r=0}^{3}\exp\left(r\alpha_{i}\theta +\delta_{ir}\right)},$$

where *δ*_*is*_ is a category-specific parameter with *δ*_*i*0_ ≡ 0 for identification purposes. With this extension the model for the item score is the generalized partial credit model Muraki (1992).^2^ Here, the conditional difficulty given no hint use is equal to (*δ*_*i*1_ − *δ*_*i*2_), and the conditional difficulty given hint use is $-\delta_{i3}/3$ .

The model in Equation 9 implies positive correlations between the item scores from different items (*S*_*i*_ and *S*_*j*_, *∀i*, *j*) because all of them are positively correlated with the latent ability. It also implies positive correlations between the response accuracies on different items (*X*_*i*_ and *X*_*j*_, *∀i*, *j*). However, it does not necessarily imply positive correlations between the hint use variables on different items (*Y*_*i*_ and *Y*_*j*_, *∀i*, *j*). Hint use variables on items *i* and *j* are positively correlated under the model when the response accuracy on these items is the same, but negatively correlated if one item response was correct and the other was incorrect. This relationship is derived from the fact that given a correct response, *Y*_*i*_ is negatively related to the latent ability *θ*:

(10) $$\text{Pr}\left(Y_{i}=1|\theta ,X_{i}=1\right)=\text{Pr}\left(S_{i}=2|\theta ,S_{i}\in \left\{2,3\right\}\right)=\frac{\exp\left(-\alpha_{i}\theta +\left(\delta_{i2}-\delta_{i3}\right)\right)}{1+\exp\left(-\alpha_{i}\theta +\left(\delta_{i2}-\delta_{i3}\right)\right)};$$

while given an incorrect response, *Y*_*i*_ is positively related to *θ*:

(11) $$\text{Pr}\left(Y_{i}=1|\theta ,X_{i}=0\right)=\text{Pr}\left(S_{i}=1|\theta ,S_{i}\in \left\{0,1\right\}\right)=\frac{\exp\left(\alpha_{i}\theta +\delta_{i1}\right)}{1+\exp\left(\alpha_{i}\theta +\delta_{i1}\right)}.$$

**Figure 2.**
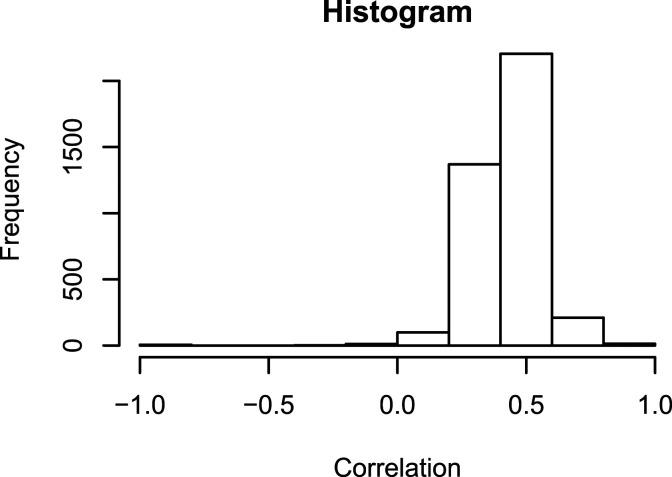
Histogram of the tetrachoric correlations between hint use variables on different items in the Spanish-from-English Duolingo data set. Most of the correlations are positive and some of them are relatively high, which means that it might be beneficial to extend the scoring-rule-based model with an additional dimension which would account for these correlations.

While the model does not generally imply positive correlations between *Y*_*i*_ and *Y*_*j*_, the tetrachoric correlations between hint use variables in the Spanish-from-English Duolingo data set are generally positive, see the histogram of these correlations in Figure 2. Therefore, it might be beneficial to extend the model to allow for these kind of correlations between the hint use variables. To account for these positive correlations, we extend the model with an additional latent variable related to the individual differences in hint use: Learners with high levels of this latent variable tend to use hints on most of the items, while learners with low levels of this latent variable tend to respond to the items without using hints. The multidimensional nominal response model (Takane & De Leeuw, 1987; Thissen & Cai, 2016) can be used to model the response accuracy and hint use where these two variables relate to the measured ability through the same scoring rule as in the model in Equation 9, and additionally the hint use variable is related to the additional latent variable:

(12) $$\text{Pr}\left(S_{i}=s|\theta ,\eta \right)=\frac{\exp\left(s\alpha_{i}\theta +ℐ\left(s\in \left\{1,2\right\}\right)\lambda_{i}\eta +\delta_{is}\right)}{\sum_{r=0}^{3}\exp\left(r\alpha_{i}\theta +ℐ\left(r\in \left\{1,2\right\}\right)\lambda_{i}\eta +\delta_{ir}\right)},$$

where *η* is the extra latent variable accounting for the differences in hint use, *λ*_*i*_ > 0 is the item loading for this latent variable, and ℐ(condtion) is the identity function which takes a value of one if the condition is satisfied, and a value of zero if it is not. Here the conditional distributions of accuracy are the same as in the model in Equation 9, but the conditional distributions of hint use depend on both latent variables, ability and tendency to use hints (*η*):

(13) $$\text{Pr}\left(Y_{i}=1|X_{i}=1\right)=\frac{\exp\left(-\alpha_{i}\theta +\lambda_{i}\eta +\left(\delta_{i2}-\delta_{i3}\right)\right)}{1+\exp\left(-\alpha_{i}\theta +\lambda_{i}\eta +\left(\delta_{i2}-\delta_{i3}\right)\right)};$$

(14) $$\text{Pr}\left(Y_{i}=1|X_{i}=0\right)=\frac{\exp\left(\alpha_{i}\theta +\lambda_{i}\eta +\delta_{i1}\right)}{1+\exp\left(\alpha_{i}\theta +\lambda_{i}\eta +\delta_{i1}\right)}.$$

The model in Equation 12 can be alternatively formulated as follows:

(15) $$\text{Pr}\left(S_{i}=s|\theta ,\eta \right)=\frac{\exp\left(a_{s+1}\alpha_{i}\theta +b_{s+1}\lambda_{i}\eta +\delta_{is}\right)}{\sum_{r=0}^{3}\exp\left(a_{r+1}\alpha_{i}\theta +b_{r+1}\lambda_{i}\eta +\delta_{ir}\right)},$$

where **a** = [0, 1, 2, 3] is the scoring function for the ability latent variable, and **b** = [0, 1, 1, 0] is the scoring function for the tendency to use hints.

### Alternative scoring rules

In the previous two subsections we have taken as the starting point that the appropriate scoring rule for the ability is to assign the most points to the correct response without hints, followed by the correct response with hints, the incorrect response with hints, and finally the lowest credit is given to incorrect responses without hints. This choice was made because we used the signed-residual-time model for response time and accuracy as a source of inspiration. We hypothesized that hint use is analogous to the use of extra time in the sense that it can be considered a resource that learners can use, and it should therefore be incorporated in the scoring rule in a similar way. However, one could argue that scores for different outcomes should be assigned in a different way. Here, we consider four alternative scoring rules:

1. Incorrect responses without hints are better than incorrect responses with hints.
2. Correct responses without hints are better than correct responses with hints, but for incorrect responses there is no difference in the scoring functions for ability between the responses with and without hints.
3. Incorrect responses without hints are better than the responses with hints. The idea here is that learners with the lowest ability will not attempt to answer the items without hints, learners with a bit higher levels of ability will attempt to answer without hints, but will often fail, and the learners with high levels of ability will attempt to answer without hints and will often succeed.
4. Only correct responses without hints receive full credit, while all other options (i.e., either an incorrect response, or using a hint) receive no credit.

Each of the alternative scoring rules can be translated in the multidimensional nominal response model with different specifications for **a**. For the three alternative scoring rules, **a** is equal to [1, 0, 2, 3], [0, 0, 1, 2], [2, 0, 1, 3], and [0, 0, 0, 1], respectively, and **b** = [0, 1, 1, 0] in all of the models. The last scoring rule corresponds to the common way of modeling responses in the presence of hints (e.g., Cen et al., 2006).

### IRTree modeling approach as an alternative to the scoring-rule-based approach

**Figure 3.**
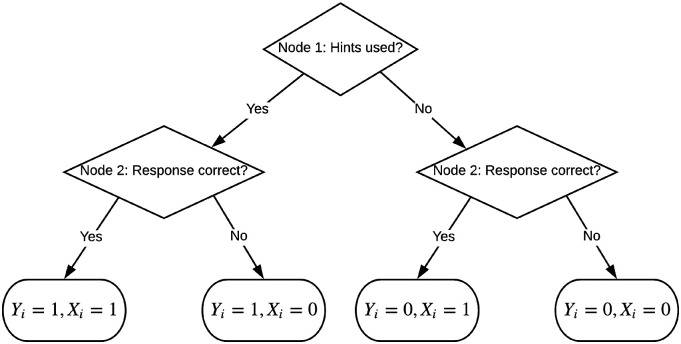
Tree-structure for responding to an item when hints are available on-demand (*Y*_*i*_—hint use, *X*_*i*_—response accuracy).

So far, we considered a scoring-rule-based approach to modeling the joint distribution of response accuracy and hint use. Alternatively, when modeling this joint distribution one might consider the observations as outcomes of two sequential events: 1. A learner decides whether to use a hint or not; 2. A learner gives a response (depending on the outcome of the first event it is a response based on the hint or no hint), which can be either correct or incorrect. Here, the joint distribution of *X*_*i*_ and *Y*_*i*_ can be factorized as the product of the marginal distribution of *Y*_*i*_ (i.e., modeling the outcome of the first event) and the conditional distribution of *X*_*i*_ given *Y*_*i*_ (i.e., modeling the outcome of the second event conditional on the outcome of the first one). This can be presented in a tree-structure (see Figure 3). Following the IRTree modeling approach of Boeck and Partchev (2012) and the generalized IRTree model of Jeon and De Boeck (2016), each node of the tree can be modeled using an IRT model. Particularly, one can model the first node of the IRTree with a 2PL model in which *Y*_*i*_ depends on the latent variable quantifying the learners’ tendency to use hints (*η*):

(16) $$\text{Pr}\left(Y_{i}=1|\eta \right)=\frac{\exp\left(\alpha_{i1}\eta +\beta_{i1}\right)}{1+\exp\left(\alpha_{i1}\eta +\beta_{i1}\right)},$$

where the intercept *β*_*i*1_ allows to model the differences between the items in terms of how often hints are used, and the slope *α*_*i*1_ quantifies the differences between the items in the strength of the relationship between the probability of a hint being used and the learners’ tendency to use hints. Analogously, for the second and the third node of the IRTree a 2PL model can also be used:

(17) $$\text{Pr}\left(X_{i}|Y_{i}=1,\theta_{1}\right)=\frac{\exp\left(\alpha_{i2}\theta_{1}+\beta_{i2}\right)}{1+\exp\left(\alpha_{i2}\theta_{1}+\beta_{i2}\right)};$$

(18) $$\text{Pr}\left(X_{i}|Y_{i}=0,\theta_{2}\right)=\frac{\exp\left(\alpha_{i3}\theta_{2}+\beta_{i3}\right)}{1+\exp\left(\alpha_{i3}\theta_{2}+\beta_{i3}\right)},$$

where *θ*_1_ and *θ*_2_ are potentially different latent variables related to the accuracy of the responses when the hints are and are not used, respectively; *β*_*i*2_ and *β*_*i*3_ are item intercepts in the second and the third node, and *α*_*i*2_ and *α*_*i*3_ are the slopes of the item in the second and the third node. Conditional on hint use, the latent variables, the item intercepts, and item slopes in the model for response accuracy can be different. Restricted versions of the model can also be considered in which some of the model elements are the same between the second and the third node.

## Methods

The set of models described in the previous section were fitted to the English-from-Portuguese data set. First, the initial model was fitted (Equation 5). Second, the model extension with the differences in discrimination was fitted (Equation 6). Third, the further model extension with the category-specific parameters *δ*_*is*_ was fitted (Equation 9), followed by the final extension with the additional latent variable (Equation 12). In the first four models, the scoring function **a** = [0, 1, 2, 3] was used. In addition to that, four multidimensional nominal response models with alternative scoring functions were fitted. Finally, five different IRTree models were fitted with the following specifications on the differences between the second and the third nodes of the IRTree:

1. *θ*_1_ = *θ*_2_, *α*_2*i*_ = *α*_3*i*_, *β*_2*i*_ = *β*_3*i*_
2. *θ*_1_ = *θ*_2_, *α*_2*i*_ ≠ *α*_3*i*_, *β*_2*i*_ = *β*_3*i*_
3. *θ*_1_ = *θ*_2_, *α*_2*i*_ = *α*_3*i*_, *β*_2*i*_ ≠ *β*_3*i*_
4. *θ*_1_ = *θ*_2_, *α*_2*i*_ ≠ *α*_3*i*_, *β*_2*i*_ ≠ *β*_3*i*_
5. *θ*_1_ ≠ *θ*_2_, *α*_2*i*_ ≠ *α*_3*i*_, *β*_2*i*_ ≠ *β*_3*i*_

The models were estimated using marginal maximum likelihood assuming a (multivariate) normal distribution of the latent variables in the population.^3^ For estimating the first two models (Equations 5 and 6) we wrote an EM-algorithm in R (R Core Team, 2018). For estimating all other models, we used the R-package mirt (Chalmers, 2012). The unidimensional models were estimated using EM-algorithm, and multidimensional models were estimated using Metropolis-Hastings-Robbins-Monro algorithm. For identification purposes, the means and the variances of the latent variables in all models except the first model were fixed to zeros and ones, respectively. In the first model, only the mean of the latent variable was constrained to zero, and the variance was estimated freely.

The models were compared using information criteria that are commonly used in the context of IRT models: Akaike Information Criterion (AIC; Akaike, 1973) and Bayesian Information Criterion (BIC; Schwarz, 1978). Furthermore, to evaluate out-of-sample prediction of the models, two-fold cross-validation was performed. The dataset was randomly split into two equal-sized subsets. Each of these subsets was treated as a testing set, while the corresponding remaining half of the data was used as a training set. All models were fitted to each of the training sets, the log-likelihood was computed for the corresponding testing set.^4^

After selecting the best models we fitted them to the full data set to obtain the final estimates of the model parameters, and also to compute the expected-a-posteriori (EAP) estimates of *θ* and *η* for each person. Furthermore, we fitted two 2PL models and obtained EAP estimates of ability under these models to compare those to the estimates of ability in the selected model. First, we considered a 2PL model for response accuracy which ignores hint use data. Second, we considered a 2PL model for data scored such that the full credit is only given to correct response without hints, and all other outcome are treated as errors.

## Results and Discussion

Table 1 shows the average AIC and BIC across the training data sets and the average log-likelihoods of each of the fitted models across the two testing sets (cross-validation log-likelihood, CVLL) of the English-from-Portuguese data. According to BIC and CVLL the best model is one of the two-dimensional scoring-rule-based models. According to the AIC the best model is the IRTree model in which the abilities, difficulties, and discriminations are different for when the hints are used or not. Given that in the context of IRT models AIC often prefers models that are too complex, we will mainly focus on the model that is chosen by BIC and CVLL. However, we also briefly discuss the IRTree model selected by AIC. In this model, the correlations between the hint-use dimension and the ability dimensions given hints and no hints were .36 [*CI*: .32, .40] and .05 [*CI*: .02, .09], respectively. The correlation between the two ability dimensions was .69 [*CI*: .67, .70]. The correlation between the intercept parameters in the hint-use node (*M* = 2.14, SD  = 1.46) and the intercept parameters in the other two nodes were −.10 [*CI*: −.29, .10] for when hints were used and −.56 [*CI*: −.68, −.41] for when hints were not used. The correlation between the intercept parameters in the second (*M* = −2.24, SD  = 0.62) and the third (*M* = −2.17, SD  = 0.65) node was .51 [*CI*: .35, .64]. None of the correlations between the slope parameters were significant (*α*_1_: *M* = 2.68, SD  = 0.81; *α*_2_: *M* = 0.23, SD  = 0.37; *α*_3_: *M* = 0.68, SD  = 0.23).

**Table 1.** Model comparison results for scoring-rule-based models with different specifications (the order of categories for ability measurement is indicated: *I*—incorrect, *C*—correct, *H*_−_—without hints, *H*_+_—with hints; “no *α*_*i*_” means that item-specific discrimination parameters are not included, “single *δ*_*i*_” means that category-specific thresholds are not included, “no *η*” means that hint-use dimension is not included) and for IRTree models with different specifications of the second (accuracy when the hint was used) and third (accuracy when the hint was not used) nodes of the tree: npar—number of freely estimated parameters, AIC—average Akaike Information Criterion in the traning data, BIC—average Bayesian Information Criterion in the training data, CVLL—average log-likelihood in the testing data. The best model based on AIC is the IRTree model with the different abilities, slopes, and intercepts when hints are and are not used. The best model based on BIC and CVLL is the two-dimensional scoring-rule-based model with highest scores for correct responses without hints, partial scores for correct responses with hints, and zero scores for all incorrect responses.

Model	npar	AIC	BIC	CVLL
Scoring-rule-based models				
*IH*_−_ < *IH*_+_ < *CH*_+_ < *CH*_−_, no *α*_*i*_, single *δ*_*i*_, no *η*	100	275,065	275,621	−137,432
*IH*_−_ < *IH*_+_ < *CH*_+_ < *CH*_−_, single *δ*_*i*_, no *η*	198	273,548	274,649	−136,867
*IH*_−_ < *IH*_+_ < *CH*_+_ < *CH*_−_, no *η*	396	241,118	243,320	−120,584
*IH*_−_ < *IH*_+_ < *CH*_+_ < *CH*_−_	496	210,563	213,322	−105,273
*IH*_+_ < *IH*_−_ < *CH*_+_ < *CH*_−_	496	210,622	213,381	−105,304
(*IH*_−_, *IH*_+_) < *CH*_+_ < *CH*_−_	496	210,522	213,280	−105,254
*IH*_+_ < *CH*_+_ < *IH*_−_ < *CH*_−_	496	210,653	213,412	−105,327
(*IH*_−_, *IH*_+_, *CH*_+_) < *CH*_−_	496	210,754	213,512	−105,361
IRTree models				
Same ability, slope, and intercept	397	211,454	213,662	−105,729
Same ability and intercept, different slopes	496	210,975	213,733	−105,543
Same ability and slope, different intercepts	496	210,545	213,304	−105,283
Same ability, different slopes and intercepts	595	210,445	213,754	−105,271
Different abilities, slopes, and intercepts	597	210,416	213,736	−105,267

For the scoring-rule-based models, each of the consecutive model extensions (adding discrimination parameters, adding extra threshold parameters, and adding the second latent variable) resulted in the improvement of model fit in the testing data and in the improvement of BIC, which means that each of these extensions appears needed to better model the joint distribution of hint use and accuracy. Among the different two-dimensional scoring-rule-based models, the model with the initial scoring rule inspired by the signed-residual-time model is not the one having the best values for the information criteria and CVLL. The best-fitting scoring-rule-based model is the two-dimensional model in which in the ability dimension incorrect responses with and without hints are not distinguished from each other in terms of scores.

In the selected scoring-rule-based model, the correlation between the two dimension was equal to .13 [*CI*: .09, .17]. That is, there is a significant positive correlation between the ability and hint-use dimensions, but this relationship is very weak (i.e., more able persons are slightly more likely to use hints). The item loadings were higher in the hint-use dimension (*M* = 2.15, SD  = 0.64) than in the ability dimension (*M* = 0.32, SD  = 0.12). This is in line with the findings of Goldin et al. (2013), who found that the individual differences in the tendency to use hints was larger than those in proficiency. Some of the items had very low loadings on the ability dimension (e.g., 0.04 and 0.09 for two of the items), which means that they are hardly contributing to the measurement of ability.

**Figure 4.**
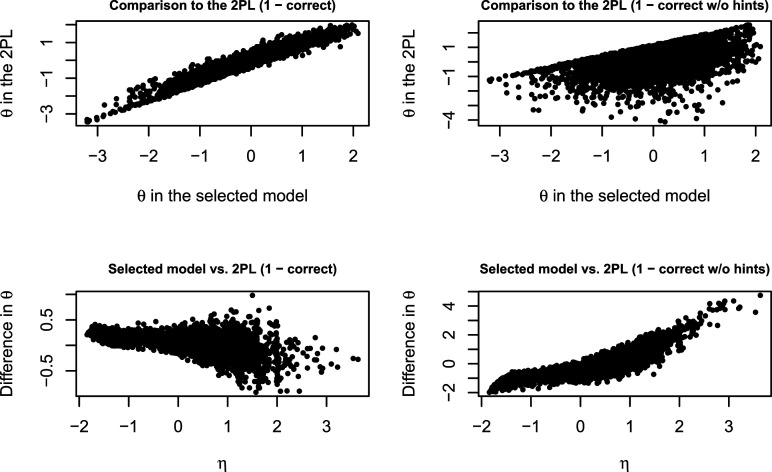
Comparison between the ability (*θ*) estimates under the selected model (two-dimensional scoring-rule-based method with full credit for correct responses without hints, partial credit for correct responses with hints, and no credit for all incorrect responses) and two-parameter logistic models (2PL) with (1) all correct responses receiving full credit (panels on the left); (2) only correct responses without hints receiving full credit (panels on the right). The upper panels show the relationships between the estimates under the two models. The estimates are a lot more similar when comparing the scoring-rule-based method with the 2PL which ignores hints use than when comparing it to the 2PL which treats hint use as incorrect responses. The lower panels show how the difference in the estimates depends on the hint-use dimension (*η*): when comparing to the 2PL which ignores hint use for the persons with low *η* the *θ*-estimate is higher for the scoring-rule-based model and vice verse for persons with high *η*; when comparing to the 2PL which treats all hint use as incorrect responses, the opposite pattern is present.

Next, we compare the estimates of ability in the selected scoring-rule-based model with estimates of ability in much simpler models in which hint-use data are either ignored, or all responses with hints are treated in the same way as incorrect responses. The upper panels of Figure 4 show scatterplots comparing the two sets of ability estimates. On the left are the abilities in the 2PL model which ignores hint-use data. They are very close to the abilities in the scoring-rule-based model, which make sense because both models give credit to all correct responses. On the right are the abilities in the 2PL model with the responses with hints coded as incorrect. For a majority of the persons, there is a notable discrepancy between the estimates under the two models. For some persons, however, there is a very strong correlation between the two sets of abilities. These are the persons who never used hints. For those persons who did use some hints, the estimates differ since in one model correct responses with hints get a partial credit while in the other they receive no credit. The lower panels of Figure 4 show how the difference between the estimates of ability in the scoring-rule-based model and each of the 2PL models depends on the estimate of the hint-use latent variable. On the left, one can see that for the persons with low hint-use the estimate of ability is higher for the scoring-rule-based model, while for high hint-use the estimate of ability is generally higher for the 2PL with all correct responses receiving full credit, but there is also more variance in the difference between the estimates. These differences are due to the fact that correct responses with hints are penalized in the scoring-rule-based method compared to the 2PL. On the right, one can see that the higher hint-use is the larger the difference between the estimates is. That is, persons who use more hints (and also give correct responses) obtain a higher score under the scoring-rule-based methods than under the 2PL with only correct responses without hints receiving full credit.

The differences found between the ability estimates in the selected model compared to the more traditional approach of measuring ability in applications in which hints can be requested pose a question about which way of operationalizing ability is more useful in practical applications. Should only successful independent execution of a task be indicative of ability or is successful completion with some help or guidance also informative? An important question which goes beyond this paper is about the validity of the different ability measures. To answer this question, one would need to, for example, evaluate criterion validity by comparing the correlations between the different ability measures and an external criterion which in the case of our application could be a score on a standardized language test. Furthermore, evaluation of convergent validity by computing the correlations between ability measures obtained from different types of language tasks would help in deciding which measure of ability to prefer.

The exact interpretation of the second latent variable in the model remains an open question. Learners with high values of *η* use more hints than learners with lower values of *η* over and above what can be expected from their ability. However, where these differences come from can be different. For example, some learners might be using hints as a learning tool, while other learners might only give a response when they are very certain of its correctness and otherwise request a hint to avoid any errors. An important topic for further research would be the relationship between the hint-use dimension and other person variables, for example gender, age, and psychological traits. The interpretation of this latent variable also depends on the application at hand, and in particular on the consequences of making an error in a learning system (e.g., whether points are subtracted from a learner, and whether a learner is forced to repeat a lesson instead of moving on) and on consequences of asking for a hint (e.g., whether a learner has to “pay” for the hint in one way or another).

An important limitation of the current study is that the data were collected in a learning context, that is, where ability is changing, but the models assume that ability is constant. The small positive correlation, between ability and the hint dimension can be possibly explained by fact that those students who ask for more hints learn more and thus their average ability over the whole period is higher than students who ask for fewer hints and learn less. It would be interesting to apply the models in settings where no change in ability can be assumed and compare the results in terms of the relationship between the latent variables. Furthermore, the proposed models could be extended with a component that accounts for learning similar to the Additive Factor Model (Cen et al., 2006).

## Conclusion

In this paper, we have described two different modeling strategies for hint use and response accuracy data from adaptive learning and assessment systems. Starting from rather simple models, we extended them in ways that deemed relevant based on the first empirical data set used for model building and tested these different models in the second empirical data set.

In the application that was considered, the best model was the one which included hint use in the measurement of ability through assigning particular scores to different combinations of hint use and accuracy with all incorrect responses receiving no credit regardless hint use. Furthermore, there were strong individual differences in the learners’ tendency to request hints. However, in a particular application depending on the type of practice items, the type of hints available in the learning system, and on the consequences of making an incorrect response for the learner within a system, the best way of modeling hint use might be different. Furthermore, making the way in which hint use is incorporated in the measurement of ability known to the learners might also change the way in which they behave and the way in which the data should be modeled.

The modeling strategies explored in this paper have potential for further developments. The scoring-rule-based models can be further explored and modified to account for additional phenomena in the learning data (e.g., strong conditional dependence between item responses) or include a different scoring rule. The IRTree models can also be further explored incorporating different tree structures (e.g., having not only binary nodes, but nodes with multiple outcomes) and multiple item attempts. Furthermore, models can be developed that take into account not only response accuracy and hint use, but also other process data variables in one scoring rule, while at the same time taking into account additional individual differences in learners behavior as indicated by the process data. For example, based on log-file data different solution strategies can be identified and different scores can be assigned to correct responses obtained with different strategies.

## Notes

1. Note, that for some items the system allows for multiple correct translations, and some typos are recognized as correct.

2. We have switched to the notation with category intercept parameters *δ*_*is*_ instead of difficulty parameter *δ*_*i*_ because this parametrization is more convenient computationally, and because it is more common for multidimensional models which are considered in the remainder of this section

3. Multivariate normality is a common assumption for multidimensional nominal response models. Testing this assumption, evaluating the robustness of the estimation procedure to its violations, and developing estimation tools that do not make such an assumption go beyond the scope of the current paper, but are important topics for further research.

4. Different approaches to model comparison can be considered in addition to or as alternatives to information criteria and cross-validation, for example, root mean squared error, area under the receiver operating characteristic curve, and other measures of accuracy. However, since these methods are not commonly used in IRT, we leave evaluation and application of such methods for further research.

## Appendix Derivation of the Scoring-Rule-Based-Model

In order to specify the scoring-rule-based model for response accuracy and hint use, we assume that scores on different items (e.g., items *i* and *j*) from the same person are independent given ability:

(19)
$$S_{pi}⊥S_{pj}|\theta_{p},$$

which is the traditional conditional independence assumption used in most IRT models. Next, we assume that the score of a person is the sufficient statistic for ability:

(20)
$$\left(X_{p\cdot },Y_{p\cdot }\right)⊥\theta_{p}|\sum_{i}S_{pi},$$

where **X**_*p*⋅_ and **Y**_*p*⋅_ are the vectors of response accuracy and hint use of person *p*, respectively.

Similar to persons, items differ from each other: On some items, people tend to get a high score, while on other items people tend to get a low score. The model can account for such differences between items by assuming that the total score of an item (i.e., *∑*_*p*_*S*_*pi*_) is the sufficient statistic for an item difficulty parameter *δ*_*i*_:

(21)
$$\left(X_{\cdot i},Y_{\cdot i}\right)⊥\delta_{i}|\sum_{p}S_{pi},$$

where **X**_⋅*i*_ and **Y**_⋅*i*_ are the vectors of response accuracy and hint use of all persons to item *i*, respectively.

Taken together, these assumptions imply that the joint distribution of response accuracy and hint use on different items can be expressed as follows:

(22)
$$f\left(X_{p\cdot },Y_{p\cdot }|\theta_{p}\right)=\prod_{i}f\left(x_{pi},y_{pi}|\theta_{p}\right),$$

where

(23)
$$f\left(x_{pi},y_{pi}|\theta_{p}\right)=\frac{\exp\left(S_{pi}\left(\theta_{p}-\delta_{i}\right)\right)}{Z_{pi}},$$

with *Z*_*pi*_ representing a normalization factor that makes the probability density function sum to one:

(24)
$$Z_{pi}=\sum_{x=0}^{1}\sum_{y=0}^{1}\left(3x-\left(2x-1\right)y\right)\left(\theta_{p}-\delta_{i}\right).$$
